# The SaeRS two-component system regulates virulence gene expression in group B *Streptococcus* during invasive infection

**DOI:** 10.1128/mbio.01975-24

**Published:** 2024-08-19

**Authors:** Francesco Coppolino, Giuseppe Valerio De Gaetano, Cosme Claverie, Odile Sismeiro, Hugo Varet, Rachel Legendre, Angelica Pellegrini, Alessia Berbiglia, Luca Tavella, Germana Lentini, Agata Famà, Giulia Barbieri, Giampiero Pietrocola, Giuseppe Teti, Arnaud Firon, Concetta Beninati

**Affiliations:** 1 Department of Human Pathology, University of Messina, Messina, Italy; 2 Department of Microbiology, Biology of Gram-Positive Pathogens, Institut Pasteur, Université Paris Cité, Paris, France; 3 Institut Pasteur, Université Paris Cité, Bioinformatics and Biostatistics Hub, Paris, France; 4 Department of Molecular Medicine, University of Pavia, Pavia, Italy; 5 Department of Biology and Biotechnology 'Lazzaro Spallanzani', University of Pavia, Pavia, Italy; 6 Scylla Biotech Srl, Messina, Italy; University of Colorado Anschutz Medical Campus, Aurora, Colorado, USA

**Keywords:** group B *Streptococcus*, two-component systems, virulence factors, host colonization

## Abstract

Group B *Streptococcus* (GBS) is a pathobiont responsible for invasive infections in neonates and the elderly. The transition from a commensal to an invasive pathogen relies on the timely regulation of virulence factors. In this study, we characterized the role of the SaeRS two-component system in GBS pathogenesis. Loss-of-function mutations in the SaeR response regulator decrease virulence in mouse models of invasive infection by hindering the ability of bacteria to persist at the inoculation site and to spread to distant organs. Transcriptome and *in vivo* analysis reveal a specialized regulatory system specifically activated during infection to control the expression of only two virulence factors: the PbsP adhesin and the BvaP secreted protein. The *in vivo* surge in SaeRS-regulated genes is complemented by fine-tuning mediated by the repressor of virulence CovRS system to establish a coordinated response. Constitutive activation of the SaeRS regulatory pathway increases PbsP-dependent adhesion and invasion of epithelial and endothelial barriers, though at the cost of reduced virulence. In conclusion, SaeRS is a dynamic, highly specialized regulatory system enabling GBS to express a restricted set of virulence factors that promote invasion of host barriers and allow these bacteria to persist inside the host during lethal infection.

**IMPORTANCE:**

Group B *Streptococcus* (or GBS) is a normal inhabitant of the human gastrointestinal and genital tracts that can also cause deadly infections in newborns and elderly people. The transition from a harmless commensal to a dangerous pathogen relies on the timely expression of bacterial molecules necessary for causing disease. In this study, we characterize the two-component system SaeRS as a key regulator of such virulence factors. Our analysis reveals a specialized regulatory system that is activated only during infection to dynamically adjust the production of two virulence factors involved in interactions with host cells. Overall, our findings highlight the critical role of SaeRS in GBS infections and suggest that targeting this system may be useful for developing new antibacterial drugs.

## INTRODUCTION

*Streptococcus agalactiae* (also known as group B *Streptococcus* or GBS) is a leading pathogen at the extremes of age, especially causing life-threatening infections in newborns and the elderly (1, 2). GBS is also the agent of a range of diseases, including sepsis, meningitis, pneumonia, arthritis, endocarditis, and soft tissue infections in patients with diabetes and other underlying chronic diseases (2, 3). GBS isolates are classified into sequence types (STs) that can be grouped into clonal complexes (CCs). Over 95% of human infections are caused by five dominant lineages (CC-1, -8/10, -17, -19, and -23), which emerged in the mid-20th century as a result of genomic recombination, antibiotic resistance acquisition, and clonal expansion (4, 5). Despite its ability to cause infections, GBS predominantly behaves as a commensal of the gastrointestinal and genital tracts of 10%–40% of healthy adults (6). The mechanisms involved in the transition of GBS from a harmless symbiont to an invasive pathogen have not yet been fully characterized, but are thought to involve CC-specific virulence factors, allelic variants, and changes in global regulators of gene expression (7, 8). The timely expression of different sets of virulence factors is essential at all stages of infection, from intestinal and vaginal colonization to invasion of tissues, blood, and meninges.

One of the main regulatory mechanisms used by bacteria to adapt their behavior to the environment is based on two-component systems (TCSs). GBS strains have 20 conserved TCSs involved in the regulation of virulence, antibiotic resistance, or metabolism (9–11). The canonical model for TCS activity is based on the detection of an environmental signal by a transmembrane histidine kinase (HK), which autophosphorylates and then transfers the phosphate group to a cognate response regulator (RR), thereby activating a specific transcriptional response. Several TCSs are required during GBS infection, including the master regulator of virulence CovRS system (9). The CovR regulator directly represses the expression of adhesins, immunomodulators, secreted proteins, and toxins (7, 8). Notably, the CovR regulatory network shows strain-specific characteristics. The plasticity of this signaling pathway is linked to mutations in CovR-regulated promoters, regulation of strain-specific genes, and mechanisms of CovR activation involving additional TCS interacting partners (7). This regulatory evolution leads to phenotypic diversity within the species and is probably linked to the emergence of clones associated with different types of infection or host tropism.

A second TCS involved in GBS pathogenesis is the SaeRS system (9, 12). The homologous SaeRS system in *Staphylococcus aureus* is a master activator of virulence genes (13). A difference between the two microorganisms is the organization of the *saeRS* locus. In *S. aureus*, the *saeRS* genes are in an operon with the *saePQ* genes encoding small transmembrane proteins necessary to fine-tune the activities of the SaeS HK (14). In GBS, the *saePQ* genes are replaced by the gene encoding the PbsP adhesin. The transcriptome of a ∆*saeRS* mutant in the A909 GBS strain (CC7) showed a large and variable regulon depending on the growth media (12). Most interestingly, the SaeR/S system is strongly activated *in vivo* in a mouse model of vaginal colonization, leading to the overexpression of the PbsP adhesin and the BvaP-secreted virulence factor (12). The binding of SaeR to the *pbsP* promoter demonstrates direct activation (12), while *pbsP* transcription is also repressed by CovR in a strain-dependent manner (15, 16).

The PbsP cell wall-anchored protein is a conserved virulence factor necessary for vaginal colonization (12), hematogenous dissemination (15), meningitis (16), and diabetic wound infection (17). The multidomain adhesin binds to host extracellular matrix components, primarily plasminogen and vitronectin, to promote adhesion and invasion of epithelial and endothelial cells (15, 16, 18, 19). In this study, we show that SaeRS-mediated regulation of PbsP is not restricted to vaginal colonization but plays a central role in host-pathogen interactions during invasive infections. Using SaeRS inactivated and activated mutants in the CC23 strain NEM316, we show that SaeRS is required in different animal models of invasive infection. While SaeRS-dependent regulation of PbsP expression is necessary at several phases of GBS disease, the constitutive overactivation of the system also decreases bacterial virulence. Transcriptomic analysis reveals a regulatory pathway that is specifically activated *in vivo* to increase PbsP-dependent adhesion and invasion of host barriers. Overall, SaeRS is a specialized system that must be tightly regulated in space and time to promote GBS pathogenesis during invasive infections.

## RESULTS

### SaeRS is required for invasive infection

**Fig 1:**
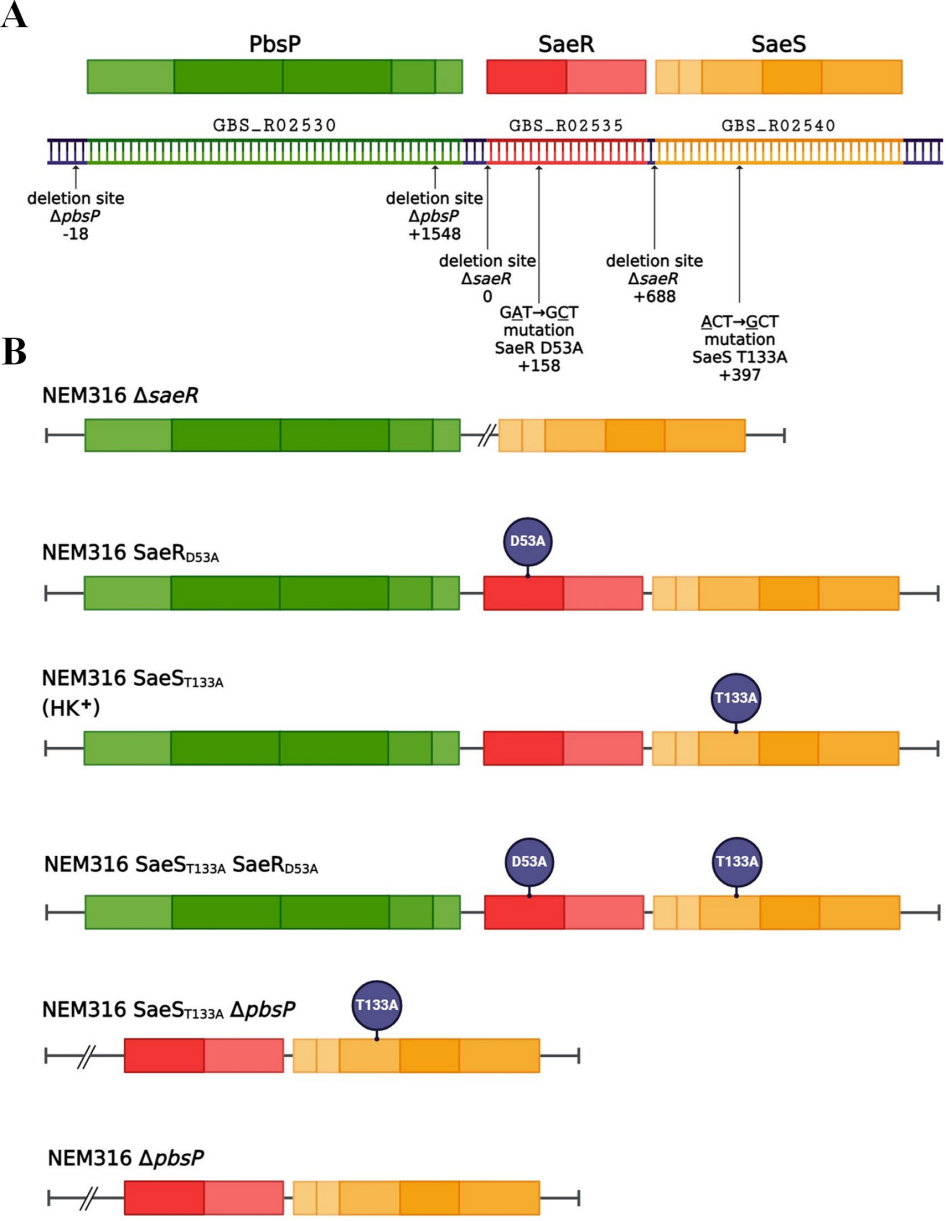
Schematic representation of NEM316 GBS mutations in the *pbsP/saeRS* locus. (**A**) Organization of the wild-type *pbsP-saeRS* genetic locus in the NEM316 chromosome and the corresponding PbsP, SaeR, and SaeS proteins with their domains. The arrows indicate the positions of deletion or point mutations in the mutant strains. (**B**) Schematic representations of each of the mutants used in this study. HK^+^, histidine kinase activated. Created with BioRender.com.

**Fig 2:**
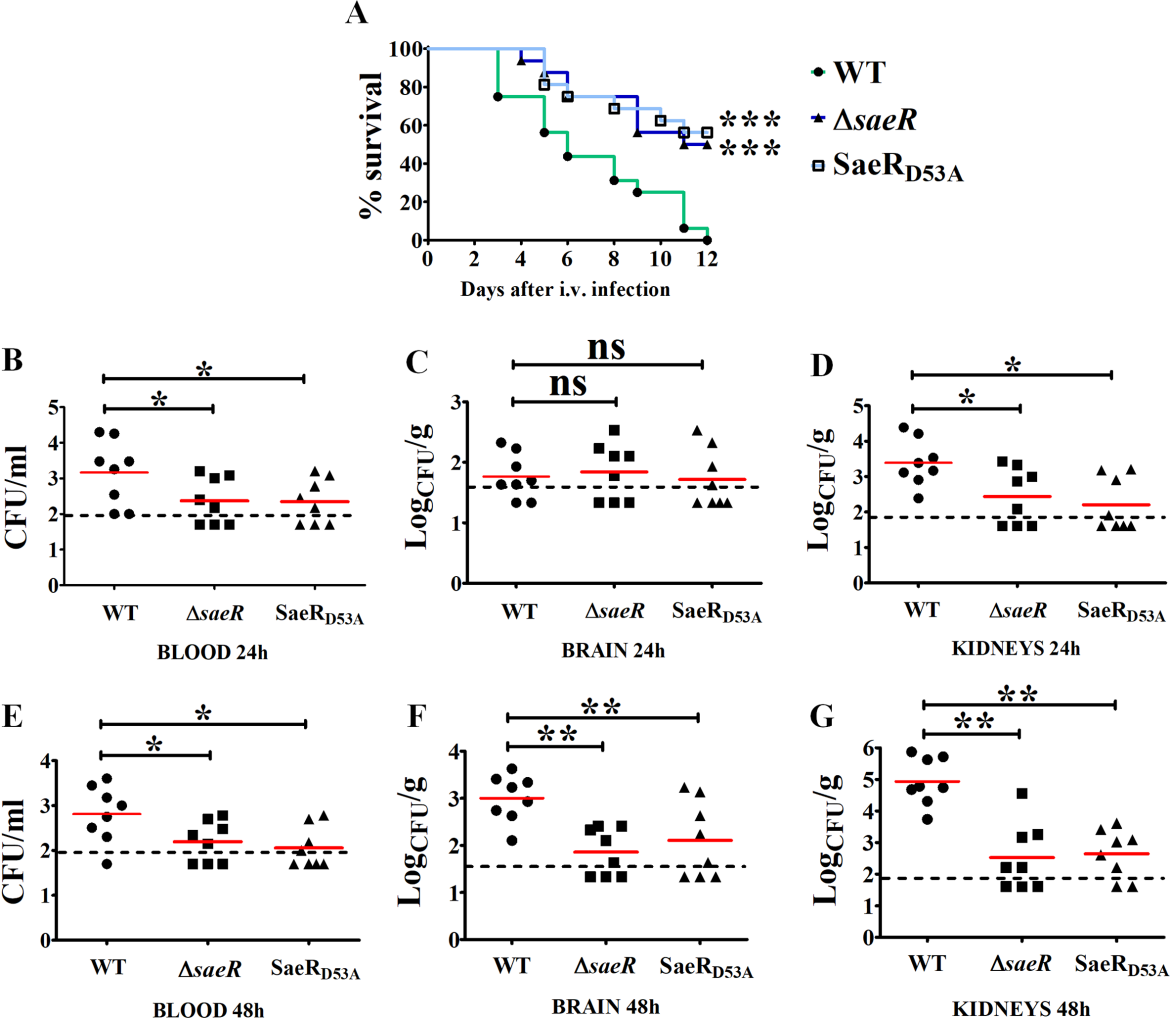
SaeR is required for virulence in a meningitis model. (**A**) Survival curves of intravenously (i.v.) infected mice. Adult female CD1 mice were infected with 10^8^ CFU of NEM316 (WT), *saeR* deletion mutant (Δ*saeR*), or SaeR-non-phosphorylable mutant with a D_53_A substitution in SaeR (SaeR_D53A_). Animals with signs of irreversible disease were euthanized. ***P < 0.001 by log-rank Mantel-Cox analysis. Shown are cumulative data from two experiments, each involving eight animals per group. (**B–G**) Effects of SaeR mutations on organ bacterial burden at 24 (**B–D**) and 48 h (**E–G**) post-infection. Shown are cumulative data from two experiments, each involving four animals per group. Horizontal red bars indicate mean values. The dashed lines indicate the limits of detection of the test. *P < 0.05; **P < 0.01; ns, non-significant as determined by the Wilcoxon test.

**Fig 3:**
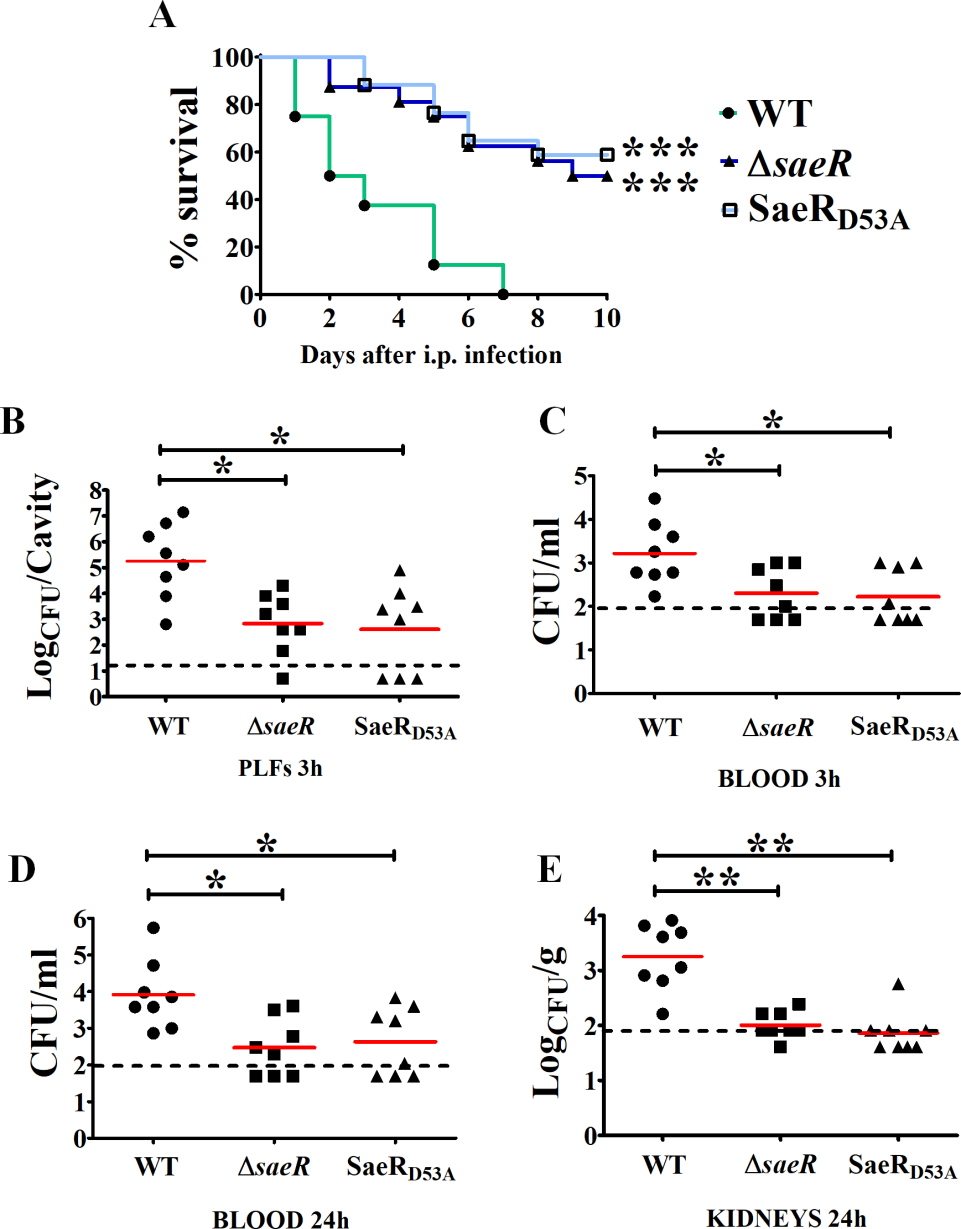
SaeR is required for virulence in a peritonitis-sepsis model. (**A**) Survival curves of intraperitonealy (i.p.) infected mice. Adult female CD1 mice were infected with 5 × 10^7^ CFU of NEM316 (WT), *saeR* deleted (Δ*saeR*), or SaeR inactive (SaeR_D53A_) mutants. Animals with signs of irreversible disease were euthanized. ***P < 0.001 by log-rank Mantel-Cox analysis. Shown are cumulative data from two experiments, each involving eight animals per group. (**B–E**) Bacterial burden in peritoneal lavage fluid samples (PLFs) at 3 h (**B**), in the blood at 3 (**C**) and 24 (**D**) h, and in the kidneys at 24 h (**E**) post-infection. Mice were infected as indicated in panel **A**. Shown are cumulative data from two experiments, each involving four animals per group. Horizontal red bars indicate mean values. The dashed lines indicate the limits of detection of the test. *P < 0.05; **P < 0.01, ns, non-significant, as determined by the Wilcoxon test.

To analyze the role of the SaeRS regulatory system in GBS, we first used loss-of-function mutations constructed in the NEM316 (CC-23) wild-type (WT) strain. The Δ*saeR* is an in-frame deletion of the *saeR* gene, while the SaeR_D53A_ mutant has a single chromosomal polymorphism (GAT→GCT) leading to substitution of the conserved aspartate residue D_53_ by an alanine, thereby preventing SaeR phosphorylation by SaeS (Fig. 1) (13). The *in vitro* growth and morphology of the two mutants and the WT strain are similar in rich media (Fig. S1A and B). To assess the role of the SaeRS system during infection, we infected mice intravenously in a model of GBS meningoencephalitis. Less than half of the mice inoculated with ∆*saeR* or SaeR_D53A_ mutants died, while all animals infected with WT GBS succumbed to infection (Fig. 2A). Quantification of bacteria in the organs of infected mice 24 h after challenge shows significantly lower bacterial counts in the blood and kidneys for both mutants compared to the WT strain (Fig. 2B through D). Similar lower bacterial loads are observed in blood at 48 h after infection, while kidney and brain invasion by *saeR* mutants is severely compromised (Fig. 2E through G), indicating that the SaeR regulator is necessary for systemic invasion. To test a second model of invasive infection, we intraperitoneally infected mice in a peritonitis-sepsis model of GBS infection. Both *saeR* mutants are less virulent than the WT strain (Fig. 3A) and are recovered in significantly lower numbers in the peritoneal cavity at 3 h post-infection (Fig. 3B). The *saeR* mutants do not efficiently spread systemically, as observed by reduced bacterial load in the blood at early time points and confirmed at 24 h post-infection (Fig. 3C through E), suggesting that SaeRS is critical for the initial phase of infection.

### SaeRS regulates specialized virulence factors

**Fig 4:**
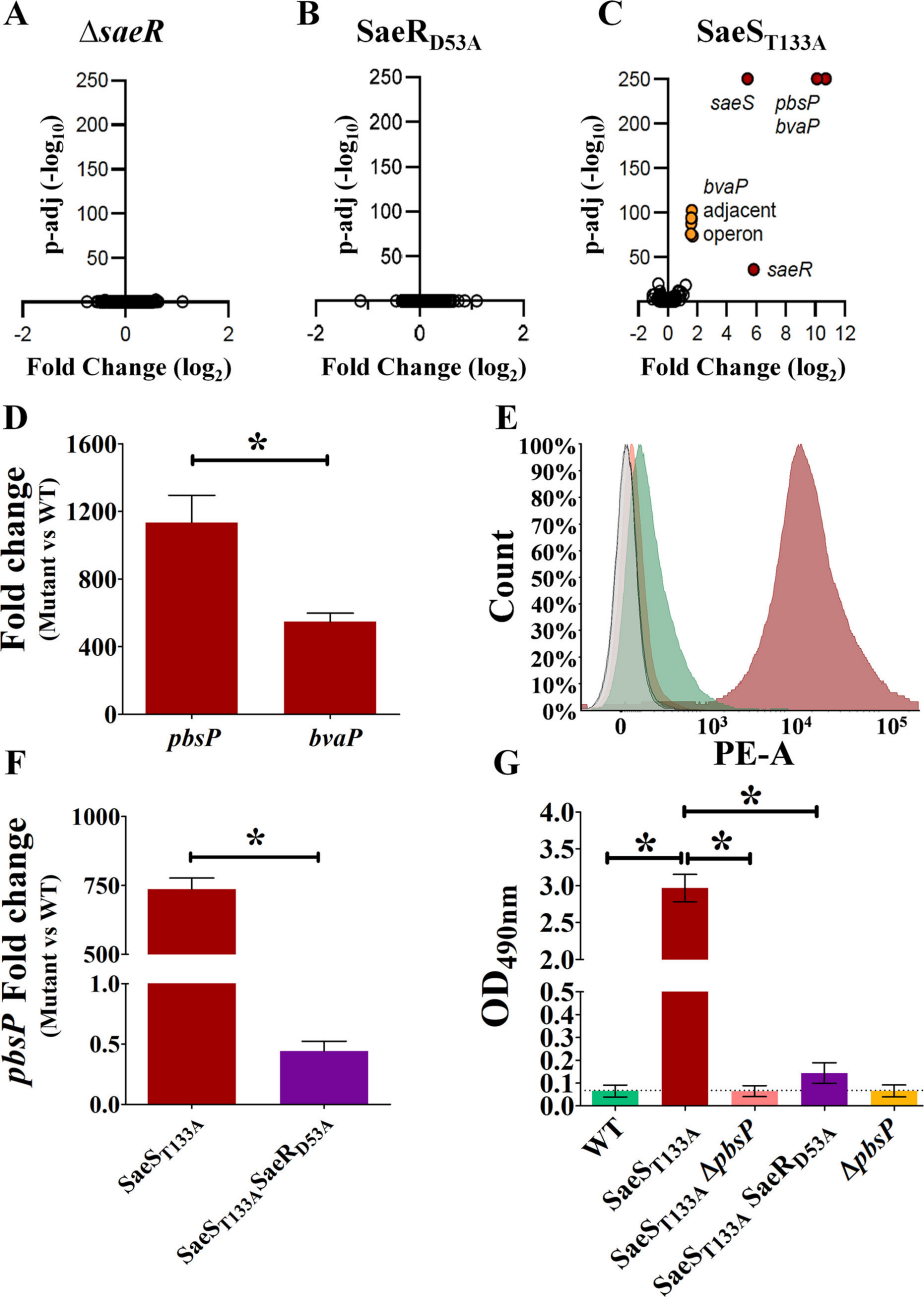
SaeRS specifically regulates the PbsP and BvaP virulence factors. (**A–C**) The SaeRS regulon. *In vitro* transcriptomic analysis by RNA-seq of the Δ*saeR* deletion mutant (**A**), the SaeR_D53A_ inactive mutant (**B**), and the SaeS_T133A_ genetically activated mutant (**C**). RNAs were purified from exponentially growing bacteria in THY at 37°C. Each dot represents a gene with its fold change relative to the WT strain and the corresponding adjusted *P*-value. Significantly regulated genes are color-coded (red: *saeRS*, *pbsP*, and *bvaP;* orange: *bvaP* adjacent operon). (**D**) Independent RT-qPCR validation of *pbsP* and *bvaP* overexpression in the genetically activated SaeS_T133A_ mutant. Results are means ± SD from three independent experiments performed in triplicate. *P < 0.05, determined by Mann-Whitney statistical analysis. (**E**) Expression of PbsP on the GBS surface. Immunofluorescence flow cytometry analysis of PbsP expression on NEM316 WT (green area), SaeS_T133A_ (red area), and SaeS_T133A_ with a deletion of *pbsP* (pink area) using mouse polyclonal anti-PbsP serum. The gray area refers to the reactivity of each GBS strain with normal serum. (**F**) SaeR phosphorylation is required for *pbsP* overexpression. RT-qPCR analysis of *pbsP* mRNA levels in SaeS_T133A_ and SaeS_T133A_ SaeR_D53A_ mutants. Results are means ± SD from three independent experiments performed in triplicate. *P < 0.05, as determined by Mann-Whitney statistical analysis. (**G**) SaeR phosphorylation is required for PbsP expression on the bacterial surface. Anti-PbsP mouse serum was used to measure PbsP surface expression using an enzyme-linked immunosorbent assay test on NEM316 WT, SaeS_T133A_, SaeS_T133A_ Δ*pbsP*, SaeS_T133A_ SaeR_D53A_, and Δ*pbsP* strains.

To identify SaeRS-regulated genes, we performed RNA sequencing of the Δ*saeR* and SaeR_D53A_ mutants. Strikingly, no significant differences in gene expression were detected in either mutant compared with the parental strain after growing bacteria in THY (Fig. 4A and B). This shows that the SaeRS system is not active in the WT strain in the tested condition. To overcome the requirement for the activating signal, we adopted a genetic approach called HK^+^ (20). This approach relies on specific inactivation of the phosphatase activity of the histidine kinase to constitutively activate the signaling pathway independently of the signal (21, 22). For SaeS, we introduced a single nucleotide polymorphism in the chromosome (ACT→GCT) leading to substitution of the catalytic threonine residue T_133_ by an alanine. Transcriptome analysis of the SaeS_T133A_ mutant revealed the highly specialized regulon of SaeRS (Fig. 4C). The regulon includes auto-regulation of the *saeRS* operon and activation of genes encoding for the virulence factors PbsP (15) and BvaP (12, 23). We also observed moderate, but significant, activation of the operon downstream of the *bvaP* gene, which is likely due to terminator readthrough in the presence of massive *bvaP* transcription (Fig. 4C). Transcriptional hyperactivation of *pbsP* in the SaeS_T133A_ mutant was confirmed by independent RT-qPCR (Fig. 4D). The overactivation of *pbsP* is corroborated by PbsP overexpression at the bacterial surface as shown by flow cytometry immunofluorescence analysis using PbsP polyclonal antibodies (Fig. 4E). Inactivation of SaeR by introducing the D_53_A mutation in the SaeS_T133A_ mutant abolishes *pbsP* and *bvaP* upregulation (Fig. 4F), confirming that the effects of the T_133_A mutation in SaeS depend on activation of the SaeR regulator.

### SaeRS is activated during *in vivo* infection

**Fig 5:**
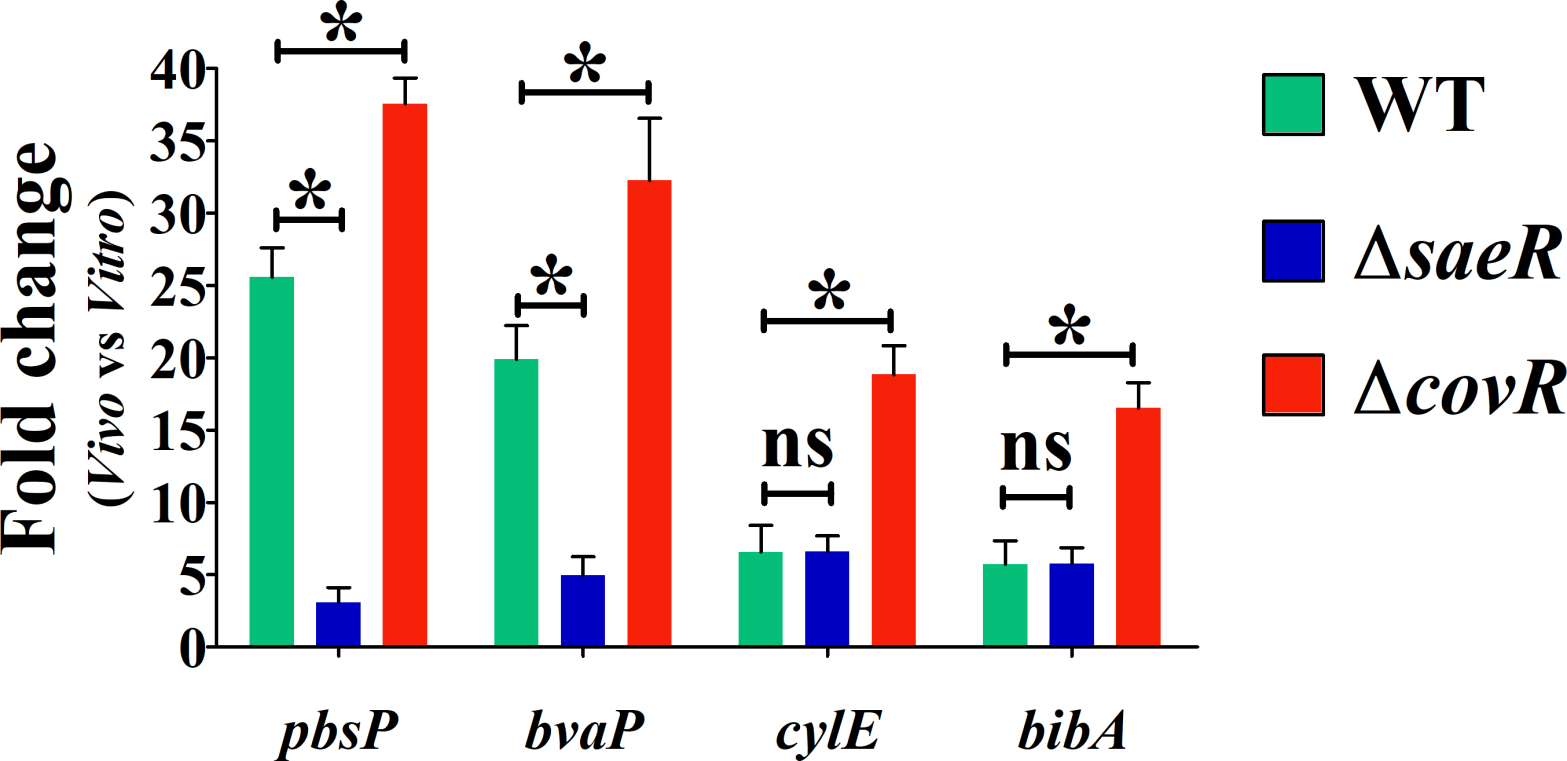
SaeRS is the main regulator of *pbsP* and *bvaP* expression *in vivo*. Transcription of virulence factors is quantified by RT-qPCR from bacteria recovered in PLF 1 h after intraperitoneal infection of mice and compared to *in vitro* growing bacteria. Transcription of positively SaeR-regulated genes (*pbsP, bvaP*) and negatively CovR-regulated genes (*cylE, bibA*) in NEM316 WT strain (green), ∆*saeR* (blue), and ∆*covR* (orange) mutants is normalized against *gyrA*, and expressed as fold change between *in vitro* and in *vivo* growth. Results are means ± SDs from three independent experiments performed in triplicate. *P < 0.05; ns, non-significant, as determined by Mann-Whitney statistical analysis.

To test SaeRS activation *in vivo*, we quantified *pbsP* and *bvaP* transcript levels by RT-qPCR in WT bacteria recovered from the peritoneal exudates of i.p. infected mice. The *pbsP* and *bvaP* genes are significantly upregulated 20- to 30-fold relative to *in vitro* grown bacteria (Fig. 5). To ascertain whether *pbsP* and *bvaP* expression is regulated *in vivo* by SaeRS, we infected mice with the Δ*saeR* mutant. RT-qPCR analysis on bacterial RNA extracted from peritoneal lavage fluid (PLF) samples indicated that *pbsP* and *bvaP* expression is markedly reduced in the Δ*saeR* mutant compared with the WT strain (Fig. 5), indicating that the SaeRS system is mainly responsible for the *in vivo* upregulation of *pbsP* and *bvaP*.

The *pbsP* gene was previously shown to be repressed by the CovR master regulator of virulence in the NEM316 strain (15). To compare SaeR-positive and CovR-negative regulations, we included as controls the *cylE* and *bibA* genes, which are directly regulated by CovR and encode for an enzyme required for the synthesis of the ß-hemolysin/cytotoxin and the BibA adhesin, respectively (24, 25). Increased transcription of the *cylE* and *bibA* genes in the WT and Δ*saeR* mutant recovered from PLF samples confirms *in vivo* activation of the CovR regulon (i.e., the release of CovR repression) in the two strains compared to *in vitro* growth (Fig. 5). We next infected mice under the same conditions using a Δ*covR* mutant and recovered total bacterial RNA from PLF samples. As expected, *cylE* and *bibA* transcription is up-regulated due to *covR* deletion and, interestingly, *pbsP* and *bvaP* transcription is also upregulated compared to the WT strain (Fig. 5). The moderate increase in *pbsP* and *bvaP* mRNA levels observed in the absence of CovR aligns with the slight but significant *in vivo* upregulation of these genes observed in the ∆*saeR* mutant in comparison with *in vitro* grown bacteria (Fig. 5). These findings demonstrate activation of both regulons during infection and confirm the co-regulation of PbsP and BvaP by primary SaeR activation and secondary CovR repression.

### Dynamic SaeRS modulation is essential for infection

**Fig 6:**
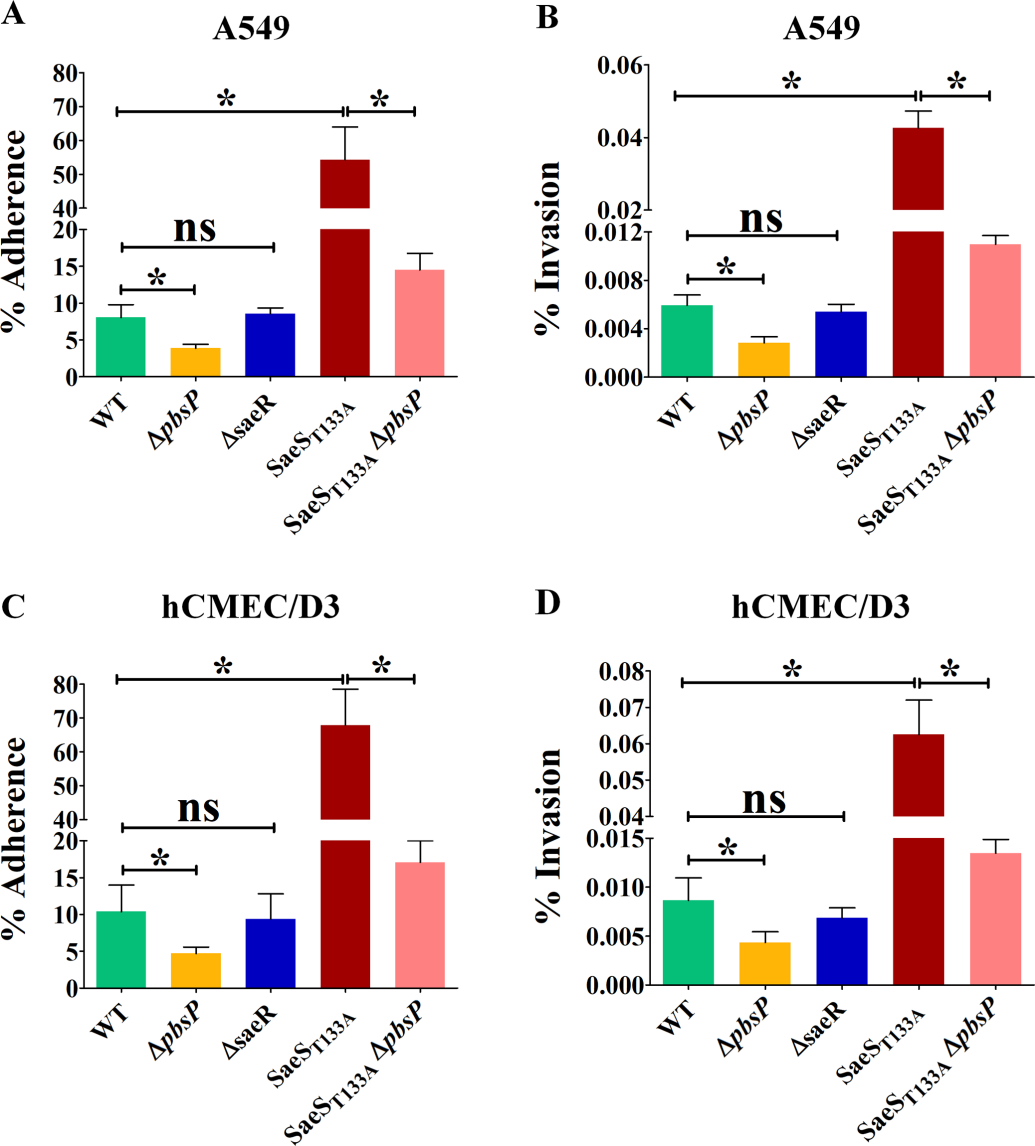
Activation of SaeRS signaling increases PbsP-dependent adhesion and invasion of epithelial and endothelial cells. (**A and B**) Adhesion and invasion of A549 epithelial cells by NEM316 WT strain (green), ∆*pbsP* (yellow), ∆*saeR* (blue), SaeS_T133A_ (dark red), and SaeS_T133A_ ∆*pbsP* (pink) mutants. Adherence and invasion are expressed as percentages of cell-associated bacteria relative to the total number of bacteria added to the monolayers. (**C and D**) Similar to panels A and B using hCMEC/D3 endothelial cells. Results are means ± SD from three independent experiments performed in triplicate. *P < 0.05; ns, non-significant, as determined by Mann-Whitney statistical analysis.

SaeRS is activated *in vivo*, necessary for virulence, and positively regulates only two virulence factors. To further characterize the role of SaeRS, we tested the activated SaeS_T133A_ mutant for host-pathogen-related phenotypes. *In vitro*, the SaeS_T133A_ mutant exhibits hyper-adhesion and hyper-invasion of A549 pulmonary alveolar epithelial cells and hCMEC/D3 brain endothelial cells compared to the WT strain (Fig. 6). To evaluate the role of the PbsP adhesin in these interactions, we repeated experiments with a ∆*pbsP* mutant constructed in the activated SaeS_T133A_ mutant. Deletion of *pbsP* in the SaeS_T133A_ mutant restores near WT levels of adhesion and invasion in both cellular models (Fig. 6). In agreement with SaeRS being inactive *in vitro*, deletion of *saeR* in the WT strain does not influence adhesion and invasion. By contrast, deletion of *pbsP* in the WT strain decreases adhesion and invasion by a twofold factor, in agreement with the presence of basal levels of PbsP expression in WT bacteria dependent on a secondary regulation. Residual, low-level adherence of *pbsP*-deleted mutants is likely sustained by the activities of adhesins other than PbsP (15, 23). Overall, these results demonstrate that the activation of SaeRS is a major determinant of host cell adhesion and invasion through positive regulation of expression of the PbsP adhesin.

**Fig 7:**
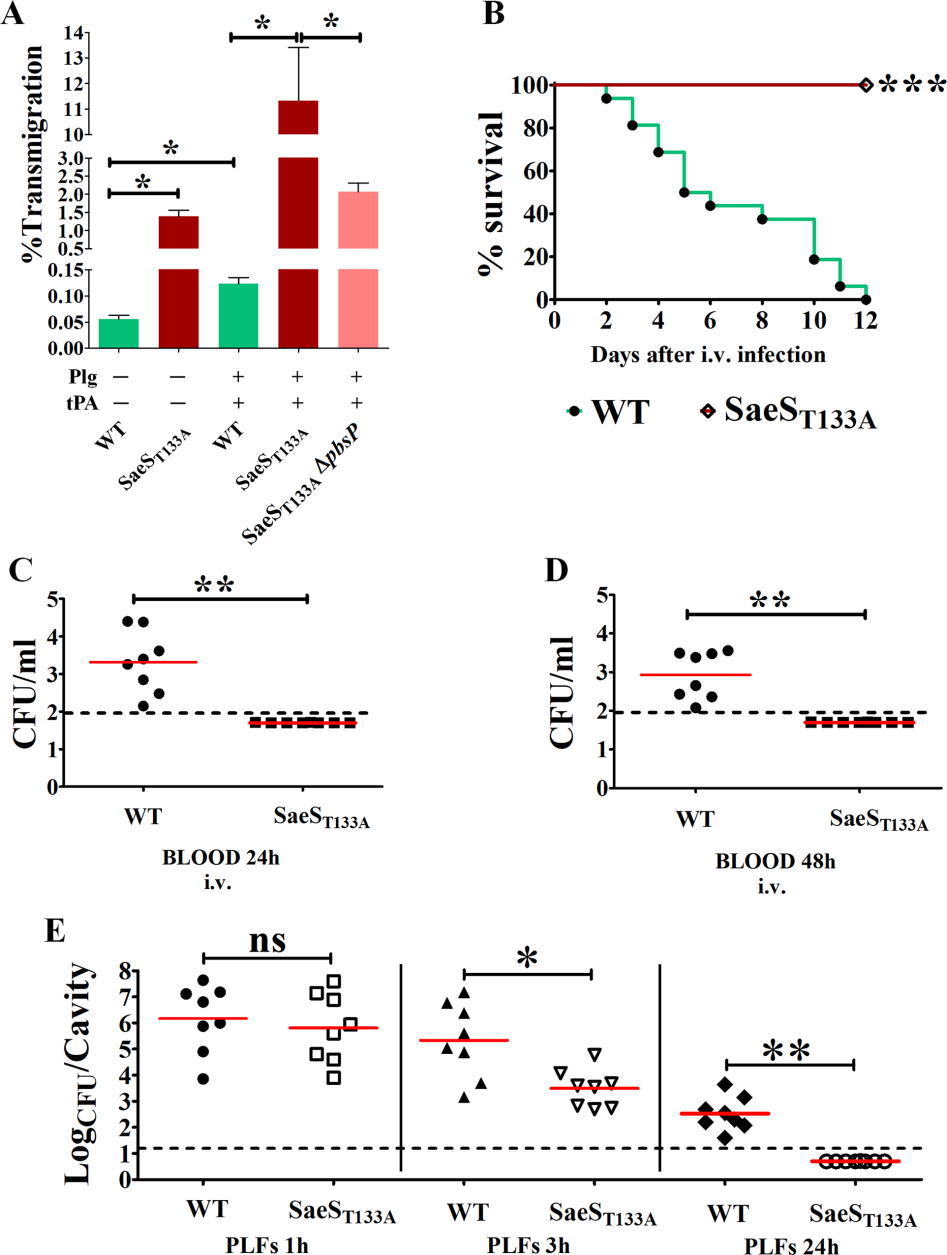
Effects of constitutive SaeR activation on *in vitro* transmigration through endothelial barriers and virulence. (**A**) GBS transmigration through brain endothelial hCMEC/D3 monolayers. Transmigration is expressed as percentages of bacteria crossing the endothelial monolayer relative to the initial number of bacteria added to the monolayer in a transwell assay in the presence or not of plasminogen (Plg) and tissue Plg activator (tPA). Wild-type NEM316 (green), SaeS_T133A_ (red), and SaeS_T133A_ Δ*pbsP* (pink) strains were tested. Results are means ± SD from three independent experiments performed in triplicate. *P < 0.05, as determined by Mann-Whitney statistical analysis. (**B**) Survival curves of mice infected with WT (green) and SaeS_T133A_ mutant (red) GBS. Adult female CD1 mice were infected i.v. with 1 × 10^8^ CFU and clinical signs were monitored. Animals with signs of irreversible disease were euthanized. ***P < 0.001 by log-rank Mantel-Cox analysis. (**C and D**) Bacterial burden in the blood 24 and 48 h post-infection. Mice were infected i.v. as in panel **B**. (**E**) Bacterial burden in PLF samples at the indicated times after i.p. challenge with 5 × 10^7^ CFU. Shown are cumulative data from two experiments, each involving four animals per group. Horizontal red bars indicate mean values. The dashed lines indicate the limits of detection of the test. *P < 0.05; **P < 0.01, ns, non-significant, as determined by the Wilcoxon test.

Increased adhesion and invasion could enhance virulence by facilitating cellular translocation across crucial defensive barriers such as the blood-brain barrier (BBB). Previous studies have demonstrated that PbsP binds plasminogen (Plg), enabling GBS to migrate across endothelial cells following the conversion of Plg to plasmin by tissue plasminogen activator (tPa) (15, 19). To investigate the role of SaeRS in GBS transmigration across endothelial cells, we utilized an *in vitro* BBB model involving hCMEC/D3 monolayers grown on transwell membrane inserts and bacteria pre-treated with Plg and tPa (16). Under these conditions, the activated SaeS_T133A_ mutant crosses monolayers much more efficiently than the WT strain, a process dependent on the overexpression of PbsP (Fig. 7A). These findings suggest that the activation of SaeRS can increase virulence by promoting epithelial adhesion, invasion, and BBB crossing. However, the constitutively activated SaeS_T133A_ mutant is avirulent when directly injected into the bloodstream (Fig. 7B). Accordingly, the SaeS_T133A_ mutant is rapidly cleared from the circulating blood (Fig. 7C and D). Similarly, the SaeS_T133A_ mutant is recovered in significantly lower numbers in the peritoneal cavity 1–3 h after intraperitoneal infection and is cleared after 24 h (Fig. 7E). The capsular polysaccharide enables GBS to evade host defenses and is, therefore, a major determinant of the ability of these bacteria to persist *in vivo* (3). However, it is unlikely that the impaired virulence observed in SaeS_T133A_ GBS is linked to decreased capsule expression since the *cps* operon is not differentially expressed in this mutant compared to the WT strain (Fig. 4C). Collectively, our data indicate that constitutive upregulation of SaeRS increases cellular invasion *in vitro* but decreases overall virulence, suggesting that dynamic regulation is necessary during *in vivo* infection. This underscores the importance of tightly regulating SaeRS activation, particularly the expression of PbsP, to promote interactions at epithelial and endothelial barriers while avoiding continuous over-activation that impedes bacterial virulence.

## DISCUSSION

The present study establishes the SaeRS two-component system as a key regulator of host-GBS interactions. This system is specifically activated during *in vivo* infection and has a necessary role in several models of invasive GBS disease while being relatively silent during *in vitro* growth. However, non-physiological, constitutive activation of the SaeRS system decreases GBS virulence, highlighting the need for tight and dynamic regulation during different phases of pathogenesis. Originally, SaeRS was shown to be activated by a small molecule (<3 kDa, probably a heat-labile peptide) present in the vaginal fluid (12). The necessity for SaeRS regulation in several models of invasive infections, as shown here, suggests the presence of a widespread activating molecule that is not exclusive to the vaginal environment. The requirement for SaeRS at several stages of infection is consistent with the pronounced *in vivo* upregulation of the adhesin PbsP and its contribution to hematogenous dissemination (15), meningitis (16), and infection of diabetic wounds (17). Taken together, our study establishes SaeRS as a dynamic system that is specifically activated *in vivo* to rapidly upregulate virulence genes and promote invasive infection.

In addition to being highly dynamic, the SaeRS system displays a remarkable degree of specialization, being specifically dedicated to the positive regulation of the cell-wall-anchored adhesin PbsP and the secreted protein BvaP. Previous *in vitro* transcriptomic analysis suggested that SaeRS regulates a large regulon of 301 to 466 genes depending on the growth media (12). However, absent or low-level *in vitro* activities severely limit functional analysis using SaeRS deletion mutants. For instance, we did not identify a single, significantly regulated gene when using *in vitro* inactivated *saeR* mutants of the NEM316 strain. To decipher SaeRS signaling, it is therefore necessary to activate the signaling pathway, either in the presence of the activating signal or by genetic engineering. By analyzing a ∆*saeR* mutant during *in vivo* growth in the mouse vaginal tract, the regulation of both *pbsP* and *bvaP* by SaeRS was previously demonstrated in the context of differential regulation of approximately one-third of the GBS genome (12). Our genetic approach bypasses the requirement for the signal and provides a focused, high-resolution view of the regulon during *in vitro* growth in rich media. We have recently systematically applied the HK^+^ approach in GBS to demonstrate the versatility of this gain-of-function strategy that works outstandingly well for SaeRS in deciphering TCS signaling (20).

The PbsP and BvaP virulence factors are positively regulated by SaeRS but are also repressed by the master regulator of virulence CovRS. One characteristic of the CovRS system is its plasticity, leading to strain-specific regulation (7). For example, CovR negatively regulated PbsP expression in the NEM316 strain (15), but this regulation was severely attenuated in the BM110 strain (16), a CC17 isolate representative of the hypervirulent lineage, although CovR binding to the *pbsP* promoter is conserved in both strains (7). In addition, overexpression of PbsP activated CovRS signaling in BM110, suggesting that PbsP acts as a signaling molecule connecting SaeRS and CovRS regulation in CC17 strains (20). We do not observe such activation of CovRS signaling in the SaeS_T133A_ mutant in NEM316, likely due to an already significant activation of CovRS signaling in the NEM316 wild-type strain compared to BM110 (7). Overall, the SaeRS and CovRS regulatory pathways are connected through CovR repression of *pbsP* and activation of CovR signaling by PbsP. This is at variance with the inability of PbsP overexpression to trigger CovRS signaling in NEM316, as found here, which might be linked to differences in basal levels of CovR activity between the two strains (7). Overall, our results show that SaeRS is the main regulator of PbsP expression and acts as a strong activator, while CovRS is a secondary repressor that fine-tunes the expression of the adhesin. The SaeRS system appears necessary to enhance interactions with epithelial and endothelial cells during mucosal colonization and invasive infections, as observed here and in a previous study (12), while CovRS globally regulates bacterial pathogenicity. Interestingly, *in vitro* PbsP expression in WT strains is variable among isolates (15), raising the possibility that the equilibrium between SaeRS and CovRS influences the infectivity and colonization potential of each strain. It is interesting to note, in this respect, that the BvaP adhesin, whose SaeRS-dependent expression also contributes to vaginal colonization, contains a variable number of repeated domains (23), suggesting selective pressure exerted by the host in altering the colonization potential of each strain.

In conclusion, we demonstrate the important role of the SaeRS pathway during systemic infection, the specialization of its restricted regulon in promoting adherence to and invasion of cellular barriers, and the presence of a dynamic regulatory network that involves, in addition to SaeRS, negative regulation by CovRS and contributes to strain specificity. This regulatory logic ensures that infection events occur efficiently, likely through initial overexpression of the PbsP adhesin and tight regulation of its production during subsequent phases of the infectious process.

## MATERIALS AND METHODS

### Bacterial strains and mutagenesis

We used the GBS strain NEM316, a human prototype serotype III clinical strain belonging to clonal complex 23 (CC23), throughout the present study. NEM316 GBS and its mutants (Table S1) were cultured in Todd Hewitt Broth (Difco, BD) supplemented with 5 g/L of yeast extract (THY; BD) at 37°C. *E. coli* strains were cultured in Luria Bertani broth (BD) supplemented with erythromycin (150 µg/mL) at 37°C with shaking. Purification of GBS genomic DNA and *E. coli* plasmid DNA was carried out with, respectively, the DNeasy Blood and Tissue kit and the Quiaprep Spin Minipreps kit (both from Qiagen), following the manufacturer’s instructions. The oligonucleotides used for genomic and transcriptomic analysis (provided by Eurofins MWG Operon) are listed in Table S2. PCRs for cloning and sequencing were performed using a high-fidelity polymerase (Phusion Plus DNA Polymerase; Thermo Scientific; cat. F630S). The pG1_Δ*saeR*, pG1_SaeR_D53A_, pG1_ΔSaeS_T133A_, and pG1_Δ*pbsP* plasmids are listed in Table S3 and were constructed using splicing by overlap extension method with primers indicated in Table S4 (15). After transforming GBS with pG1_Δ*saeR*, pG1_SaeR_D53A_, pG1_SaeS_T133A_, or pG1_Δ*pbsP*, integration, and de-recombination events were selected as described (15, 20). The presence of the desired mutations was confirmed by whole-genome sequencing using the Illumina MiSeq platform. The Δ*covR* strain was described in a previous study (26).

### Animal models of GBS infection

Virulence of GBS was tested in 6- to 8-week-old CD1 female mice (Charles River) in accordance with the European Union guidelines for the use of laboratory animals. In the meningitis model, mice were infected i.v. with ~1×10^8^ bacteria in a total volume of 0.1 mL of Dulbecco’s PBS (DPBS, Sigma-Aldrich) and clinical signs were monitored every 12 h for 12 days. Animals with signs of irreversible disease or neurological signs, as assessed using a scoring system (27), were humanely euthanized. In a second group of experiments, i.v. infected mice were sacrificed at 24 or 48 h after infection to collect blood, brains, and kidneys. Organs were homogenized in the gentleMACS dissociation system (Miltenyi Biotec), as previously described (16, 28). The number of colonies forming units (CFU) was measured in organ homogenates using previously described methods (15). In the sepsis model, CD1 mice were intraperitoneally (i.p.) injected with ~5×10^7^ CFU in 0.2 mL of DPBS and monitored every 12 h for clinical signs as detailed above. GBS replication in the peritoneal cavity and its systemic spreading to other tissues was verified by plating blood, organ homogenates, and peritoneal lavage fluids (PLFs) at different time points. PLF samples were obtained by injecting 2 mL of phosphate-buffered saline (PBS) in the peritoneal cavity and subsequently aspirating a total of 1.7–1.9 mL of fluid, as previously described (29–32).

### Quantitative RT-PCR and RNA sequencing

To measure the transcriptional levels of genes encoding for GBS virulence factors, bacterial RNA was extracted from PLF samples obtained at 1 h post-infection or from bacteria grown *in vitro* in THY, retro-transcribed, and analyzed using real-time PCR (RT-PCR), exactly as previously described (16). After PLF collection and centrifugation (12,000 × *g* for 10  min), eukaryotic cells were lysed by exposure to cold distilled water for 10 min. To collect bacteria, tissue debris and residual eukaryotic cells were removed by low-speed centrifugation (200 × *g* for 10 min) and, subsequently, supernatants were centrifuged at high speed (12,000 × *g* for 10 min) to obtain the bacterial pellet. Quantitative PCR (qPCR) was performed with the Taqman Gene Expression Master MIX (Applied Biosystem, cat. 4369016) using probes (shown in Table S2) to detect the following transcripts of the following genes: *pbsP*, *bvaP*, *cylE*, *bibA,* and *gyrA* by the CFX Opus Real-time PCR System (Biorad). Relative gene expression levels were calculated with the ΔΔCT method, where expression values were normalized with the expression of the housekeeping *gyrA* gene. Each experiment was performed in triplicate.

RNA extraction, purification, sample processing, and data analysis for RNA-seq were done as described (7, 20). Briefly, a biological triplicate of exponentially growing bacteria in THY at 37°C (OD_600_ = 0.5) is centrifuged, washed with cold PBS containing RNA stabilization reagents (RNAprotect, Qiagen), and mechanically lysed by bead-beating (Precellys Evolution, Bertin Technologies) in RNApro reagent (MP Biomedicals), before RNA purification by chloroform extraction and ethanol precipitation. Residual genomic DNA is removed (TURBO DNase, Ambion) and RNA quantified and their quality validated (Qubit RNA HS, Invitrogen; Agilent Bioanalyzer 2100) before ribosomal rRNA depletion (FastSelect Bacterial, Qiagen) and libraries construction and sequencing following the manufacturer’s instructions (TruSeq Stranded mRNA, NextSeq 500, Illumina).

Single-end strand-specific 75 bp reads were cleaned of adapter sequences and low-quality sequences (cutadapt version 1.15) and only sequences at least 25 nt in length were considered for further analysis. Bowtie v1.2.1.1 with default parameters was used for alignment on the NEM316 genome (NCBI: NC_004368). Genes were counted using featureCounts version v1.5.3 from a Subreads package (parameters: -t locus_tag -g ID -s 1). Count data were analyzed using R version 3.6.1 and the Bioconductor package DESeq2 version 1.26.0. The normalization and dispersion estimation were performed with DESeq2 using the default parameters but statistical tests for differential expression were performed by applying the independent filtering algorithm. A generalized linear model was set to test for the differential expression between the biological conditions. For each pairwise comparison, raw *P*-values were adjusted for multiple testing according to the Benjamini and Hochberg (BH) procedure and genes with an adjusted *P*-value lower than 0.05 were considered differentially. Raw sequencing reads and statistical analysis are publicly available (GEO accession number GSE269249).

### Flow cytometry analysis

PbsP expression on the bacterial cell surface was visualized using flow cytometry immunofluorescence analysis using a mouse anti-PbsP serum and a normal serum control, as previously described (18). Briefly, GBS cells (~1×10^8^) grown to the log phase in THY were washed in DPBS, fixed with 3.7% formaldehyde, and blocked using DPBS supplemented with 1% milk for 30 min at 22°C and gentle shaking. Bacteria were incubated with the mouse anti-PbsP serum diluted 1:50 for 1 h with gentle shaking. Subsequently, a phycoerythrin (PE-A)-conjugated goat anti-mouse IgG (ThermoFisher, cat. 12-i10-82) diluted 1:50 in 1% milk was used to reveal primary antibody binding. Fluorescent bacteria were analyzed with a FACSCanto II flow cytometer using the FlowJo software (BD Biosciences).

### Enzyme-linked immunosorbent assay

For testing PbsP expression on the GBS surface, 96 well Nunc MaxiSorp flat-bottom plates (Thermo Fisher Scientific; 44-2404-21) were coated at 4°C overnight with streptococci at a density of ~1×10^7^ CFU/well in 0.05 M carbonate buffer (pH 9.5). After washing and blocking with 5% BSA in Tris-buffered saline pH 7.5 (TBS; 50 mM Tris-Cl; 150 mM NaCl), PbsP expression was evaluated using mouse anti-PbsP serum diluted 1:4,000 in TBS-1% BSA and incubated for 1 h at room temperature (RT) with gentle shaking. To detect antibody binding, anti-mouse IgG conjugated with horseradish peroxidase (HRP) diluted 1:1,000 in TBS-1% BSA was added and left for 45 min at room temperature. After the addition of o-phenylenediamine dihydrochloride (ODP; code 34006, Thermo Scientific), absorbance at 490 nm was determined in an enzyme-linked immunosorbent assay (ELISA) plate reader.

### Adhesion and invasion

The human cell line A549 (type II alveolar epithelial cells, ATCC CCL-185) was grown in MEM/F12 medium consisting of a 1:1 mixture of F-12 medium (ATCC 30-2004) and Eagle’s minimum essential medium (EMEM; ATCC 30-2003), supplemented with 10% (vol/vol) fetal bovine serum (FBS, 1203C, Sigma-Aldrich). The human brain endothelial cell line hCMEC/D3 (brain microvascular endothelial cells, kindly provided by P.O. Couraud, INSERM, Paris, France) was grown in Endothelial Cell Medium 2 (C-22011, Promo Cell), supplemented with SupplementMix (C-39216, Promo Cell) and 10% FBS at 37°C in a humidified 5% CO2 incubator. Adherence and invasion assays were performed exactly as previously described (33, 34). For the invasion assay, the monolayers were washed and incubated with a medium supplemented with penicillin and streptomycin (200 U/mL and 200 µg/mL, respectively) to kill extracellular bacteria, as previously described (35). Percentages of bacterial adhesion and invasion were calculated as recovered CFU/initial inoculum CFU × 100.

### GBS migration assay

To test the transmigration ability of CC23 NEM316 and *saeR* mutant strains, an *in vitro* model of the endothelial blood-brain barrier was established by cultivating hCMEC/D3s on collagen-coated polycarbonate transwell membrane inserts with a pore size of 3 µm (Corning), as previously described (15, 16). This model mimics GBS penetration through the blood-brain barrier and consists of an upper and a lower chamber corresponding to the “blood side” and the “brain side,” respectively. The inserts were placed in 12-well plates and the hCMEC monolayer was allowed to grow until confluence in the upper chamber while the bottom chamber was filled with 1.5 mL of fresh growth medium. Prior to the assay, the integrity of the cell monolayer was confirmed by adding Evans blue to the upper chamber. Before infection, hCMECs were rinsed and left in a serum-free culture medium without antibiotics. GBS, untreated or treated with 50 µg/mL of human plasminogen plus 20 nM tissue plasmin activator (tPA), was applied to the apical chamber at a multiplicity of infection of ~20, exactly as described (16). Next, the number of bacteria crossing the endothelial monolayers was enumerated by plating the lower chamber medium at 2 h post-infection.

### Statistical analysis

Survival data were analyzed by log-rank Mantel-Cox analysis and differences in bacterial CFU counts were assessed by the Wilcoxon test. All the remaining data relative to the other assays were analyzed by the Mann-Whitney test. Differences were considered statistically significant when *P* values were less than 0.05.

## Data Availability

Raw sequencing reads and statistical analysis of RNA sequencing data are publicly available (GEO accession number GSE269249). All other data will be made fully available by the corresponding author upon reasonable request.

## SUPPLEMENTAL MATERIAL

Fig. S1 mbio.01975-24-s0001.docx Growth curves of GBS mutant strains.

Supplemental Tables mbio.01975-24-s0002.xlsx Tables S1 to S4.

ASM does not own the copyrights to Supplemental Material that may be linked to, or accessed through, an article. The authors have granted ASM a non-exclusive, world-wide license to publish the Supplemental Material files. Please contact the corresponding author directly for reuse.
