# Supplementary material for: The SaeRS two-component system regulates virulence gene expression in group B Streptococcus during invasive infection
Source: mBio. 2024 Aug 19;15(9):e01975-24. doi: 10.1128/mbio.01975-24 (PMC11389388; doi:10.1128/mbio.01975-24)
Supplement: Fig. S1 — Growth curves of GBS mutant strains. [file mbio.01975-24-s0001.docx]

**Fig. S1**


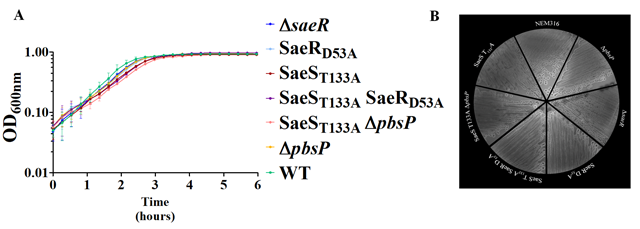


**Fig. S1. Growth curves of GBS mutant strains**. (**A**) Growth of wild-type NEM316 (WT), Δ*pbsP* and *saeRS* mutant strains in THY medium, evaluated as absorbance at OD_600_ (y axis) in the course of time (x axis). (**B**) SaeRS mutations do not affect hemolytic activity in GBS. Representative image of β-haemolysis in wild-type, Δ*pbsP* and *saeRS* mutant strains grown on blood agar. The clear halos surrounding bacterial colonies (black dots) on blood plates correspond to lysed erythrocytes.
